# Experiences and Perceived Barriers of Asylum Seekers and People with Refugee Backgrounds in Accessing Healthcare Services in Romania

**DOI:** 10.3390/healthcare10112162

**Published:** 2022-10-29

**Authors:** Liliana Dumitrache, Mariana Nae, Alina Mareci, Anca Tudoricu, Alexandra Cioclu, Alexandra Velicu

**Affiliations:** Faculty of Geography, University of Bucharest, 010041 Bucharest, Romania

**Keywords:** refugees, asylum seekers, transit country, healthcare system, barriers, Romania

## Abstract

Traditionally a country of emigration, Romania recently experienced an increased migration influx, although it is more a country of transit than a destination for refugees and irregular migrants. Refugees often face difficulties when trying to meet their needs and access essential services. This study aims to explore the experiences and barriers of asylum seekers and people with refugee backgrounds in accessing healthcare services in Romania. It is an exploratory study with a qualitative research design, which uses an inductive and deductive approach, with thematic analysis being applied in order to grasp the difficulties and barriers that asylum seekers and people with refugee backgrounds experience in accessing essential social services. The research was based on seven in-depth interviews with representatives of significant national or international non-governmental organisations assisting refugees and asylum seekers in Romania and 129 semi-structured interviews with different categories of people with refugee backgrounds from Southwest Asia, Eastern Africa and Ukraine. One of the most salient themes we identified relates to accessing the healthcare system. Participants talked about what information they needed to access medical services, where they looked for this information, and what barriers they faced in the process. Cultural, linguistic, structural, and financial barriers were perceived as the most significant. Improved public awareness, a better understanding of asylum issues, and stronger community support are essential to addressing inequalities experienced by this vulnerable population.

## 1. Introduction

The growing intensification of flows, the mass transit migration, the emergence of new migration routes, and new stages in migratory trajectories framed the political and social agenda during the past years. Despite this, the dynamic of travel from origin to destination, the transit regions and spaces, and the relocation and resettlement processes are still insufficiently understood and explored. Irregular migrants, refugees, and asylum seekers are challenging groups with disruptive societal shifts.

In recent years, Europe has experienced an increased influx of migrants in vulnerable situations. In 2015, over 1 million Syrian refugees fleeing war-related violence arrived in Europe. The continent faced a crisis of governance and showed the inadequacy of protective measures and border control enforcement. Since the “refugee crisis” moment in 2016 and the appeal to solidarity by the members of the European Union, scholars emphasised differences in refugee protection by national authorities [1,2], international and EU solidarity deficiencies, and limits of municipal solidarity with refugees [3,4]. Gaps from a jurisdiction perspective still exist between member states. International cooperation arrangements failed to ensure a functional refugee system since they were considered outside the jurisdiction of states belonging to the Global North, while less developed countries from the Global South offered hospitality centres for many refugees [5,6].

Member States needed to offer refugee protection (due to EU asylum acquis) and solidarity. However, since the 2015–2016 “refugee crisis”, Europe took restrictive measures and laws, shifted responsibility outward, and reinforced border control [7,8,9]. Other countries such as Turkey and Libya shared the responsibility for refugees’ reception through multilateral agreements. Turkey hosted Syrian citizens in equipped camps at nine sites attempting to provide necessary assistance, particularly healthcare services [10,11]. However, significant literature pointed out the shortcomings of the existing conditions, highlighting their negative impact on the refugees’ wellbeing [12]. All over Europe, authorities are making efforts to engage local municipalities to integrate refugees and asylum seekers into communities.

The concepts of “transit migration” and “country of transit” were mainly used by institutions and international organisations without an agreed definition [13,14]. “Country of transit” is considered a rhetorical expression used by politics and non-governmental organisations (NGOs), a contested space, a space of “temporariness” [15]. Refugees seek to settle in a country or just pass through. In the latter case, interactions between natives and refugees can happen for a short time and are highly transient. However, reactions in locals differed from increased anxiety since the first arrival of refugees to their settlements [16], changing voting behaviour, to negative media coverage of the refugee crisis [17,18]. Among many other reasons, fear was proven to be the “standpoint of rejection” [19].

At the EU level, under the concept of “country of transit”, all countries must cooperate and coordinate their actions in the form of “mobility partnerships”, externalisation of border control, and shifting responsibility for preventing irregular migration into Europe onto the transit country [20]. In September 2020, the European Commission launched the New Pact on Migration and Asylum with a package of legislative proposals. A “voluntary and temporary” solidarity mechanism would eliminate mandatory relocation quotas. Once activated, other EU countries would offer their assistance by hosting a share of the migrants in their territory, sponsoring the return of those whose applications to stay inside the EU had been rejected, and providing financial contributions and operational support to the country/countries under pressure [21].

Another step followed in June 2022, when the European Union started the first stage of policy reform on asylum and migration. The plan is to modernise the asylum seeker and illegal migrant database. In addition, 21 countries have adopted a declaration providing a solidarity mechanism to support frontline Member States [22].

In 2021, the number of people displaced across national borders hosted in European countries surpassed 7 million, representing an increase of 3% compared to the previous year. This increase reflects newly recognised refugees—288,000 persons—located mainly in Germany (79,700), France (51,000), and Italy (21,100). However, Turkey remained the largest refugee-hosting country in the world in 2021, with more than 3.8 million refugees at year-end, or 15% of all people displaced across borders globally [23].

In Europe, the arrival of refugees and migrants continued to increase, presently including the influx of Ukrainian refugees. The latest figures, available from April to June 2022, showed that 447,784 refugees and migrants arrived in frontline countries (Greece, Italy, Bulgaria, and Serbia), including 378,736 refugees from Ukraine and 44,956 arrivals from Asia (mainly from Afghanistan, Bangladesh, Pakistan, Syria, and Iran) and North African countries [24].

Romania is already known as a typical country of emigration, and immigration is considered relatively low. However, after its EU admission in 2007, the country started to receive inward migration, with a total of 137,000 foreign citizens registered in 2021, of which approximately 80,000 were citizens from non-EU countries. Furthermore, due to its geographical position, Romania became an alternative destination for migrants and refugees, although most only transit here on their journey to Germany, France, or Spain. Thus, the number of refugees in Romania remains reduced—4200 in 2021 [25]—and the resettlement of refugees is considered low—109 Syrian refugees from Turkey and Jordan, representing the 2018–2019 quota [26].

According to the United Nations High Commissioner for Refugees (UNHCR), 9558 asylum applications were received in 2021 in Romania; most of them came from Afghanistan, Syria, and Bangladesh. People who arrive fleeing a war context are granted refugee status—a form of protection recognised by the Romanian state to foreign citizens or stateless persons who meet the conditions set out in the Convention relating to the Status of Refugees concluded in Geneva on 28 July 1951, referred to as the Geneva Convention, to which Romania acceded as per Law No. 46/1991 [27].

The start of the war in Ukraine in February 2022 caused the displacement of millions of persons: As of 18 September, 1,246,945 Ukrainian citizens crossed the border into Romania, of which more than 80,000 remained [24]. According to the UNHCR and Romanian General Inspectorate for Immigration, up to 14 August 2022, a total of 54,899 temporary protection residence permits were issued, and 4345 asylum requests from Ukrainian citizens were registered; in other words, approximately 1 in 10 displaced persons from Ukraine chose to settle in Romania [28]. This makes Romania one of the top 10 destination countries, on par with Slovakia, the Republic of Moldova, and Bulgaria, but well below Poland, Germany, Turkey, the Czech Republic, Italy, and Spain [29]. Poland remains the largest receiver, primarily due to its proximity but also the strong and historical economic relationships between the two countries [30]; between February 24 and 11 April 2022, over 2.7 million Ukrainian and third-country nationals entered Poland as a result of the war [31].

The crisis in Ukraine has also prompted the Romanian state to take steps to protect and support the resulting refugees. The Emergency Ordinance No. 20, published on 7 March 2022, detailed support and humanitarian assistance measures, thus providing solutions to ensure the rights to education, healthcare, work, protection for children and persons with disabilities, the right to receive free medical assistance and appropriate treatment through the national system of emergency medical assistance and qualified first aid, integration of minors into the Romanian education system or access to the unemployment insurance system, measures to prevent unemployment, and measures for stimulating employment [32].

Efforts initiated by NGOs and municipalities also serve to stymie some of the more extreme exclusionary policies. Since the conflict in Ukraine, Romania has received operational support in the form of an estimated 160 national and international NGOs and 2000 volunteers. At the national level, over 1200 refugee centres hosted 11,000 people, and they provided assistance to over 660,000 refugees [33].

The regular accommodation centres for asylum seekers in Romania are subordinated to the General Inspectorate for Immigration. They are organised in six Regional Centres of Procedures and Accommodation (Bucharest, Galați, Radăuți, Șomcuta Mare, Giurgiu, and Timișoara). Separate from people coming from Ukraine, most asylum seekers who applied for humanitarian protection in Romania came from Syria, Iraq, Pakistan, Afghanistan, and Algeria [34].

Romania represents a focal point of the Western route of the Balkans. However, it did not significantly impact the migration map in 2015–2016 [35]. Previous studies highlighted that refugees consider Romania a transit country, merely a passage route to Western Europe. Efforts for their integration have been made, and while most participants mentioned some drawbacks of settling in Romania, the country was still seen as an accommodating place and an alternative or a better choice compared to containment camps located on the Mediterranean seaboard [36]. This is not singular to Romania, as studies have shown neighbouring countries as not final destinations but merely transitional ones. In the context of international migration, neighbouring countries have similar migration characteristics in terms of country of transit along the Balkan Route [37].

## 2. Access to Healthcare for Asylum Seekers and Refugees: Focus on Romania

Refugees, asylum seekers, and undocumented migrants are displaced populations that encounter, during their journey, social risks, insecurity, and violence with undesirable and detrimental health outcomes [38]. The nature of research on migration and health is heterogenous since it is generally difficult to assess the impacts on health outcomes during the different stages of migration (pre-migration, movement, arrival, transition, integration, or return stages). It is necessary to emphasise the dynamic relationships between migrant typology and various health outcomes in order to understand the association between migration and health. This is because refugees, asylum seekers, and undocumented migrants are subjects of different levels of health vulnerabilities and social isolation; they reside in or pass through unsecured spaces such as derelict urban zones, construction sites, and transport corridors [39]. They carry several health burdens, occupy different social positions, go through distinct migration experiences, and face barriers as they seek access to health services in the host destination. Poor living conditions, unemployment, bureaucratic procedures, and entitlements can cause additional stress [40].

They are considered vulnerable groups due to their challenging experiences before and during the displacement and the arrival in the host country. However, since vulnerability is a multi-layered concept, refugees should not be considered vulnerable per se; each person might be assessed on situational fragilities and needs [41]. Therefore, barriers and challenges to accessing healthcare need to be pointed out. Relevant qualitative healthcare studies focused on barriers and facilitators to accessing care [42], the responsiveness of the healthcare professionals when providing medical assistance to refugees [43], policy implications for optimising the initial healthcare of refugees [44], access of refugees to informal interpreters, and the use of primary and secondary care services [45].

Feldmann et al. [46] found that positive relationships need to be developed, and general practitioners must invest empathy and trust and avoid negative attitudes or actions based on prejudices. Communication skills and personal behaviour of health personnel are considered crucial in meeting patient needs, and guidance materials are necessary to understand the cultural background of these social groups [47].

In addition, because of the nature of their forcible displacement, this population is exposed to socio-cultural and economic factors acting as stressors related to healthcare issues, lack of education opportunities, community support, and insecurity about their legal status.

Socio-cultural and economic factors act as barriers that can hinder the utilisation of suitable and basic necessities, creating a mismatch between service needs and service utilisation. As Pap and Glied [48] noted, sometimes ”religious and cultural approaches clash with the pragmatic demands” (p. 106). Previous research underlined barriers related to missing knowledge about services, lack of access to information [49], educational attainment [50], medical care and the absence of health insurance coverage [51,52], and linguistic and cultural problems. Socio-cultural barriers are generally understood as the dissonance between the cultural background of the host destination compared to the country of origin or a cultural mismatch between refugees and asylum seekers and the host population [53,54,55,56,57].

Access to healthcare is limited by legal entitlement or the structure of the new country’s health system, which varies across European countries in terms of regulation and laws [58].

Across the WHO European Region, the ability of the state to provide healthcare for refugees and asylum seekers varies according to the development of health service infrastructure and the funding of healthcare for the general population [59] as well as asylum seekers and people with refugee backgrounds unfamiliarity with the healthcare system and networks of healthcare professionals [60,61].

After the Syrian crisis, healthcare provisions for refugees have fallen mainly to civil society, involving teams of volunteers that have sprung up in many European countries and the longer-established international humanitarian agencies [61]. NGOs can take longer-term views, work innovatively and flexibly, operate in contested spaces, and gain the trust of those who have come to fear government officials [62]. For example, they played a big part in providing healthcare in Greece and work alongside government institutions in Italy, Slovenia, and Spain [63], but their ability to provide continuity of care and use local resources such as general practitioners and nurses is less certain [59]. Complementary to the healthcare system, education is a key to socio-economic success and overcoming disadvantages [64]. It offers opportunities to rebuild social networks and fosters a sense of coherence, agency, and hope, having been placed next to housing and employment in third place for facilitating integration [65]. On the other side of the spectrum, researchers showed that lower levels of education result in increased separation and marginalisation [65,66,67,68]. Fluency in the host country’s language is critical to a successful integration [69], while poor language skills and socio-economic deprivation are identified as significant obstacles.

From an economic perspective, if the refugees are allowed to work, they have the potential to contribute to the host countries as employees, taxpayers, and innovators. While some studies showed that mass transit migration had a critical negative economic outcome on entrepreneurship [70], others emphasised the positive effects on knowledge creation and innovation [71,72]. Ajzenman et al. also demonstrated that exposure to transit refugee flows had a significant effect on the entrepreneurship of the natives; nevertheless, a negative impact on institutions’ confidence and perceived political and legal stability has been identified [73]. If refugees are integrated into the labour market, they become self-reliant, and the costs of support of the host governments and the donors of hosting refugees are reduced. However, refugees in many countries do not have formal labour market access.

Romania remains a transit country for most asylum seekers and refugees planning to go to Western countries. Nevertheless, the government renewed the integration programme targeting the beneficiaries of international protection, including counselling services, support activities for access to medical and social assistance, housing, employment, social security, and education.

The Romanian healthcare system has been subject to continuous reforms after the fall of communism in 1989, and many changes have occurred at different levels. Despite reforms targeting decentralisation, this was not fully achieved, with the Ministry of Health remaining the central administrative authority [74] and dominated mainly by the public sector.

The private health network, which has expanded since 2005, is continuing to develop. Family physicians and GPs provide primary care. Clinics and hospital units offer secondary and tertiary care. Hospitals are essential components of health systems and still play a central role in the delivery of healthcare in Romania [75]. The public healthcare system is organised and financed according to the Healthcare Law 95/2000 [74]. The Romanian healthcare system is based on a mandatory health insurance scheme, which covers all Romanian citizens and foreigners legally residing in Romania. Most health funds derive from the population, predominantly through social health insurance contributions and taxation. The mandatory health insurance contribution represents 5.5% of employees’ monthly income and 5.2% of employers. People contribute to the medical system through the National Insurance Fund, and the insured beneficiaries can access a free basic package of medical services. In addition, children, students, and vulnerable citizens benefit from pay exemptions. Unfortunately, out-of-pocket payments increase the final costs of medical services.

Refugees, asylum seekers, and people under subsidiary protection have the right to receive healthcare and medical assistants as Romanians do, according to Government Ordinance no. 44/2004 regarding the social integration of foreigners who were granted a form of protection in Romania. The same Ordinance states asylum seekers’ right to work and assistance in their job search. Having the right to work makes asylum seekers eligible for health insurance if they can afford to pay the contribution [76]. Law no. 122/2006 states that individuals who seek a form of international protection are entitled, free of charge, to primary medical care and adequate treatment, emergency, hospitalisation, healthcare, and treatment in cases of acute or chronic diseases that imminently endanger their life. They also have an obligation to present themselves to the medical examinations established for them. These healthcare services are provided by the medical services of the accommodation centre or by other health units [77].

The state guarantees children access to medical services, and the costs are covered by the National Health Insurance Fund and the state budget. Children of asylum seekers are exempted from contributing to the mandatory health insurance and can benefit from it until they are 18 years old.

Evidence states that entitlements for asylum-seekers and refugees take into account integration and health concerns; they can access basic information on these entitlements through the Romanian General Inspectorate for Immigration and National Healthcare Insurance House (NHIH). Primary care and mental health services offices are established in the Regional Centres of Procedures and Accommodation. These services provide medical assistance in family medicine and reproductive and family planning [78].

Despite studies that highlight challenges and barriers for refugees and asylum seekers to healthcare in Romania [79], we need to consider the context of the humanitarian crisis since the war in Ukraine, the transient nature of the migration process, the patterns of each stage of migration, and the uniqueness of the interaction between people with a refugee background and the institutional setting authority. Therefore, our analysis is exploratory in nature, as we are not focusing on the health of refugees and asylum-seekers, but we are looking at how participants perceived barriers to accessing the healthcare system. The migration process is a changing, uncontrolled, and fortuitous situation (for example, 50,000 Ukrainian refugees crossed the Romanian border in just two days in September 2022, according to official data—it is possible that not all of these refugees interacted directly with the healthcare system and healthcare professionals).

This paper uses thematic analysis to explore how certain conditions and multiple barriers influence the halt of the migration journeys or settlement of refugees in Romania. We conducted seven in-depth interviews with representatives of various NGOs and associations and 129 semi-structured interviews with asylum seekers and people with refugee backgrounds from Southwest Asia, Eastern Africa, and Ukraine regarding their experiences and the difficulties they faced navigating our national systems. We derived two salient emerging themes, out of which access to healthcare is preeminent. This theme provides insights into the challenges and barriers this vulnerable population experienced in accessing medical services, how participants tried to solve their health-related problems, and details about their experiences during their interactions. In addition, participants talked about what information they needed to adapt to the Romanian culture, where they looked for this information, and what barriers were insurmountable.

## 3. Methods

A qualitative design was adopted for this study. Qualitative data analysis was completed using thematic analysis with a combination of deductive and inductive approaches. Thematic analysis is considered an analytical and flexible method that could offer an accessible form of data analysis that describes patterns across qualitative data. It is regarded as a flexible method and does not require a consistent and detailed theoretical framework (such as grounded theory or phenomenological epistemology) to gain, describe, and understand everyday people’s experiences. The thematic analysis can be used for different theoretical frameworks (essentialist and constructivist approaches) to reflect reality and unravel the surface of reality [80]. Semi-structured and in-depth interviews were applied to facilitate a broader exploration of the experiences of asylum seekers and people with refugee backgrounds during their accommodation in the host country.

A coding framework was manually created, and the dataset was analysed by deducing the emerging themes: Access to healthcare and access to education, of which this study focuses on access to healthcare. Asylum seekers and people with refugee backgrounds experienced difficulties accessing these services, with the main barriers being structural, financial, cultural, and linguistic. The perspective of the NGO representatives was also considered. Part of the textual and thematic analysis was performed using an open-source, web-based application: Voyant Tools v.2.4 (voyant-tools.org) [81].

Participants were recruited using purposeful/convenience and a snowball sampling method. This sampling method does not primarily focus on participants’ representativeness but rather on their knowledge and experience regarding the topic in question. Purposeful sampling is employed to ensure the “identification and selection of information-rich cases for the most effective use of limited resources”; as such, it may hamper generalising findings as it often neglects the heterogeneity of the population groups sampled [73]. We achieved data saturation, i.e., when interviews stopped yielding new information. Similar to other studies [82], our research took place in the capital city and a few major cities in the country because it was shown that these locations are dominant sites of settlement for recent arrivals, as they offer more abundant opportunities for education and employment.

The interviews were conducted following set ethical protocols. Full names and home addresses were excluded from the questions. Ethical approval for the study was issued by the University of Bucharest Ethics Committee decision no. 42/19.05.2022, following its Ethics and Deontology Code. Interviews were conducted between May and July 2022, face to face, in Romanian, Ukrainian, or English. In some cases, a translator facilitated communication. Responses from the interviews conducted with individual asylum seekers and people with refugee backgrounds were noted by an operator using a tablet in Google Forms, and a database was created. All participants agreed to an audio recording of the interviews, and the average duration for each conversation was 30 min.

The recordings were then transcribed and translated into English. The emerging themes were derived inductively from the interview data and deductively using significant concepts from relevant literature. Part of the textual and thematic analysis was performed using an open-source, web-based application Voyant Tools v.2.4 (voyant-tools.org) [81].

### 3.1. Participants

#### 3.1.1. Asylum Seekers and People with Refugee Backgrounds

We conducted 129 semi-structured interviews with individual asylum seekers and people with refugee backgrounds. Table 1 presents an overview of participants’ demographic characteristics. Most are young adults, with the dominant age group being 30–45 years, 40.6%, followed by 18–29 with 35.2%. Most respondents were under subsidiary protection (37.2%), refugees (33.3%), or asylum seekers (9.3%).

Respondents’ legal status is closely correlated with the duration of their stay in Romania and the reason for leaving their country of origin. Half have been in Romania for less than 1 year, 26.4% for 1–5 years, 10.1% for 5–10 years, and only 13.2% for more than 10 years (Table 1).

The reasons for their arrival to Romania vary; most came here because of armed conflict, 51.2%, and only 1.2% because of persecution. Almost 53% of respondents have completed a form of higher education, and just over half are employed. Those unemployed with a work permit—12.4%—share an almost equal percentage with those who are unemployed without a work permit, 15.5%. In terms of country of origin, most respondents are from Ukraine (54.3%), followed by Syria (6.2%), Iraq (6.2%), and refugees from Southeast Asia, the Middle East, and Africa. Permanent residents (16.3%) have already been granted a residence permit and have proof of a continuous and legal stay of a minimum of 5 years on national territory. Naturalised citizens have obtained Romanian citizenship, have been permanently residing in Romania for at least 8 years, and are fluent in the Romanian language. In the refugee context, however, local integration as a durable solution would imply permanent residence, meaning permanent settlement in a country of first asylum and eventually being granted the nationality of that country.

#### 3.1.2. Representants of Non-Governmental Organisations (NGOs) and Associations

We have centred our discussions on NGO representatives as their work often supersedes governmental entities in challenging anti-immigrant sentiments and protecting migrants [61]. The in-depth interviews were conducted with representatives of associations with an NGO background, some having a long history of experience in working with refugees while others with only a few months of experience; we also engaged with representatives of a specialised UN agency.

They are AIDRom (Ecumenical Association of Churches in Romania) in Bucharest and Timișoara, the Islamic Cultural Center Crescent Foundation in Bucharest, the Aluziva Association in Bucharest, the Terre des Hommes Foundation in Bucharest, JRS Romania (Jesuit Refugee Service—Galați Regional Integration Centre), and IOM (International Organisation for Migration) in Bucharest (Table 2).

### 3.2. Data Collection and Instrumentalisation

The operators who went into the field were trained for a few days before the data collection. Interview guidelines were reviewed, and sampling and logistical issues were discussed. Interviewers had prior experience and were trained in conducting open-ended interviews. Many interviews were conducted at mosques, places of prayer for the Muslim community, near accommodation centres, or in NGO representatives’ offices after obtaining appointments. Field investigations were also conducted in Siret, Rădăuți, and Dumbrăveni, small towns in Suceava County, an important crossing point on the Romania–Ukraine border. In addition, field investigations were conducted in Galați and Timișoara, both border counties, where we had meetings with representatives of AIDRom Timișoara and Galați Regional Integration Centre under the aegis of JRS Romania, but also with individual asylum seekers and people with refugee backgrounds.

The research questions for this study were addressed within a paradigmatic framework of interpretivism. We focused on the interpretive and reflexive approach to reflect migrants’ accounts of their attitudes, opinions, and experiences during their stay or temporary period in Romania. The coding and theme development processes were flexible, analytical, and reflexive and were used with a combination of inductive and deductive approaches. Through the inductive approach, the themes identified are related to the data (bottom-up way) [83], where the process of coding the data is not fitted in pre-existing coding and is considered data-driven. Using the inductive approach, within collected interviews, we read and re-read the data for any themes related to refugees and asylum seekers, and we sought to focus on a particular feature in coding the data. The deductive approach is theoretically explicit, applied in a top-down way, and considered to be analyst-driven. The essence of the theme consisted of its capacity to capture something important in the entire dataset. In our case, barriers to access to health care and education represent salient themes to understanding the meanings and experiences of this population group during their stay in a foreign country (what information they needed to understand the Romanian culture, the health and education systems and where they looked for this information, and the relationships between language and communication). Language is an implicit vehicle in the social production and reproduction of meanings and experiences. Thematic analysis evolves a reflexive approach, which is a time-consuming and iterative process. We analysed, organised, and structured corpus data following different stages: Familiarization with the data, generating initial codes, generating themes, reviewing potential themes, defining and naming the themes, analysis, and interpretation.

## 4. Results

### Barriers Asylum Seekers and People with Refugee Backgrounds Experienced in Accessing Healthcare Services in Romania

The main types of barriers were identified during our discussions with members of the target group and representatives of NGOs working with this vulnerable population. To support a general overview of healthcare barriers, the summarised results are presented in Table 3.

Overall, over half of the participants stated that no prominent obstacles had been endured during their interaction with the healthcare system and healthcare professionals. However, 48.1% of the respondents perceive communication skills and language barriers as more pronounced. Thus, 17.7% always encounter language barriers and 30.7% named language as a usual barrier. Legal, structural, and financial barriers were encountered by over 30% of the participants, while 23.3% claim a lack of community support.

Barriers and challenges to accessing the healthcare system provide insights and significant meanings in the accounts of all participants. Language barriers, low communication skills, and a general lack of knowledge of the healthcare system were deterrents to participants trying to receive healthcare services. Barriers to healthcare are various, seen as obstacles (getting to an appointment with the doctor, receiving specialised treatment, and following physicians’ instructions).

Access to the healthcare system largely depends on human resources and logistics. However, access to healthcare for asylum seekers and refugees is mostly restricted due to legal constraints, limited capacity in accommodations, inadequate information on the availability of healthcare services, language difficulties, and cultural differences between host and origin countries. Although they are in a foreign country, refugees have the right to healthcare under national and international law, as access to it is a fundamental human right. During the forced journey out of their country, refugees may face harsh and precarious conditions that severely affect their physical and psychological health. They often arrive in host countries feeling tired and lost, with language, financial, and other barriers making it difficult or even impossible to access healthcare providers and obtain the necessary services, medicines, and medical supplies.

Refugees, asylum seekers, and the representatives of the NGOs identified a mismatch between the medical system structure and functioning, the lack of financial resources, and language barriers. However, for half of the respondents in our study, accessing the healthcare system in Romania was “very easy”. Only 2.3% of respondents mentioned that this was “very difficult”. Most opinions gravitated towards the middle of the scale, with 17.1% of respondents feeling they had “some difficulty”, 27.9% considering access “easy”, and 3.9% perceiving it to be “difficult”.

Respondents perceived the language aspect as a barrier stating that there is a general communication problem due to language differences. In fact, out of those that named language as a barrier, it was “always” a barrier for 17.7% of them. Issues related to communication problems raised concerns about engagement in therapy and treatment. Many mentioned that they had difficulty communicating with hospital or pharmacy staff due to a lack of language skills on one or both parts. The medical staff’s lack of English language skills hindered hospital admissions and discussions about procedures. The fact that previous medical prescriptions and other documents had to be translated into Romanian complicated the situation for respondents, and more time was needed to resolve it.

Additionally, people from Middle Asia and Ukraine are not familiar with the Latin alphabet and therefore face difficulties in finding an appointment with their health professional services

*‘I was not medically insured, and we couldn’t understand each other because of the language. We used Google Translate’*

[Man, 30–45 years old, Ukraine]

In situations where communication is impossible or support is needed, one of the NGO members declared that they accompany the refugee/immigrant to the doctor. They translate and help with the whole bureaucratic process.

*‘I called 112 and was taken to the hospital by ambulance for appendicitis surgery. Admission was difficult due to poor language skills’*

[Woman, 46–59 years old, Irak]

In line with several studies, miscommunication with both professional healthcare workers and informal interpreters was considered an issue by asylum seekers and refugees as patients. Healthcare professionals have often faced prominent challenges with migrants or have limited experience working with them [44,84]. Professional interpreters are more proficient in medical terminology, while informal interpreters might produce poor-quality translations [85].

*‘Language is a barrier; few doctors speak English or other languages apart from Romanian. Also, a lot of them are in a rush and don’t pay enough attention (we don’t have time for you!). In my opinion, when the doctor does not cooperate well with us, we need to go to another doctor, and this really wastes our time’*

[Woman, 18–29 years old, Turkey]

Communication skills also imply time to give care and willingness to take time to listen since the patients are referring to the lack of time of paying less attention on the part of the caregivers (*We don’t have time for you!*). The patients expected to see health professionals being kind, empathetic, and enduring by using in their communication interaction process non-verbal cues to improve trust and alleviate anxiety. From the cross-sectional analysis of corpus data, participants emphasised the importance of friendliness and not creating the feeling of otherness on the side of a healthcare professional. An expression that frequently occurred in participants’ discourse was: *the need to understand each other is key*.

*‘Understanding each other. We had a lot of documents and referrals, and we had to translate everything’*

[Man, 18–29 years old, Ukraine]

Negative and critical episodes concerned long waiting lists, issues with insurance coverage, a restrictive doctor, and (dis)trust in doctors:

*‘The greatest difficulty that I had in accessing the healthcare system was when I was very sick, and I could not afford the medication because I had no health insurance. I was able to resolve it with the financial support of my family’*

[Man, 18–29 years old, Bangladesh]

The lack of resources for specialised treatment is considered a frequently perceived barrier. Respondents faced difficulties accessing waiting lists because they were full, or experienced restrictions in finding a specialised doctor when needing secondary or tertiary healthcare services.

*‘My husband was admitted to a hospital in Bucharest and the medical staff were not kind because he was a foreigner. The doctor told us that he had to consult several doctors and wait a long time for the laboratory test report. Now, he gets free treatment and the conditions in the medical system are better’*

[Woman, 46–59 years old, Pakistan]

Other structural barriers have been recurrent such as the issue related to the health system management, bureaucracy, and complex procedures. Participants did not know how to get an appointment or understand the emergency medical system. They often cite their inability to afford the indirect financial costs of care.

*‘I do not understand the system’*

[Woman, 30–45 years old, India]

*‘I was not given any support regarding the health system, being considered a foreigner and had to bear all the fees, without any kind of discount’*

[Man, 18–29 years old, Bangladesh]

*‘My children needed medical care, and I had to buy some medicines because the hospital didn’t have them, and I asked my relatives to help me’*

[Woman, 30–45 years old, Yemen]

*‘There are some people who pay for their health insurance; if they can’t afford it, we have implemented programs that can help them pay their insurance for up to 6 months’*

[B1]

Asylum seekers and people with refugee backgrounds often gave different explanations for the causes of health and illness and the utilisation of medical services. They also rarely sought out care for mental health, and many had a lack of understanding of mental health conditions. However, local and community support was beneficial for the refugee in this case.

*‘The landlady helped me find a psychologist who spoke Arabic, this process took quite a long time. Before she managed to find me a doctor who spoke Arabic so that I could understand everything, she was advising me and be able to get through this difficult stage because I’m mentally down. I don’t have a stable job, and it’s very hard for me’*

[Man, 18–29 years old, Syria]

NGO representatives mentioned several difficulties regarding the identification of family doctors. For example, family doctors started refusing to register beneficiaries of international protection, including children, because the system requires them to register patients for at least six months, and they were afraid that beneficiaries would only transit Romania. A more recent problem is the sheer number of registrations that have to be made. Another reported issue is related to health insurance. Members and volunteers of NGOs are often engaged in helping refugees fill in the necessary formalities for obtaining medical insurance.

Results of the text analysis related to the access of asylum seekers and people with refugee backgrounds to healthcare might provide interesting insights. For example, pairs of words in the corpus data revealed associations of significance relating to the experiences and relationships between patients and doctors.

**Figure 1:**
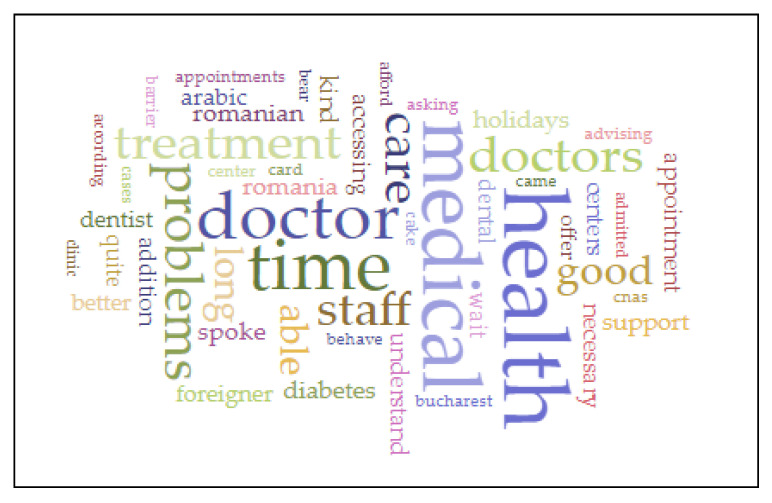
Word cloud on occurrences related to asylum seekers and people with refugee backgrounds’ access to healthcare.

To observe the similarity in text, we expect to find the same words in pieces of the text, where some words are more important than others. The significance is measured as a TF-IDF score, a common way of expressing how important a term is in a document relative to the rest of the corpus. TF-IDF (term frequency—inverse document frequency) is a numerical statistic that is intended to reflect how important a word is to a document in a collection. It is often used as a weighting factor in searches for information retrieval and text mining (voyant.tools.org). A word cloud for occurrences was created for the section of interviews from the database as the source text, with bigger font sizes showing that that issue is more frequent (Figure 1).

A textual analysis of relationships between pairs of words revealed some interesting insights. The correlation tool enables an exploration of the extent to which term frequencies vary in sync between the first term of the pair and the second term of the pair. Furthermore, Pearson’s correlation coefficients for pairs of words and the significance of the correlation value (lower is better) are displayed.

Pairs of words such as “language—support”, “care—good”, “better—health”, “doctor—time”, “necessary—treatment”, and “accessing—treatment” are associated with high Pearson’s correlation coefficients and high significance of the correlation value (Table 4).

The correlation coefficient is calculated by comparing the relative frequencies of terms (relative to each document for the corpus or relative to each segment of the document). A coefficient that approaches 1 indicates that values correlate positively; they rise and fall together. A coefficient that approaches –1 indicates that values correlate negatively; frequencies rise for one term as it drops for the other.

Two significant strands can be displayed when examining corpus data of the NGO representatives’ discourses. On the one hand, they participate actively and are involved in various social activities using strategies to help asylum seekers and people with refugee backgrounds cope, recognise citizenship, and obtain their social rights. On the other hand, survival and coping strategies are linked to resources (social networks, work, and payments for basic needs) [86,87]. As the in-depth interviews showed, in Romania, these resources, many times, are scarce or dependent on legislation that is convoluted and often changing and, more often than that, summarily implemented. Nonetheless, they shared with us the dysfunctions and exclusionary connotations of being an asylum seeker or person with a refugee background and often emphasised that when it came to people fleeing the war in Ukraine, access to the Romanian system was made easier, especially from a legislative and social support perspective.

## 5. Discussion

This study aimed to explore the experiences of asylum seekers and people with refugee backgrounds and the potential barriers that limit their access to healthcare services. Refugees and asylum seekers face various difficulties accessing essential social services in Romania.

Legal, linguistic, financial, and administrative barriers are considered major challenges when these social groups access and use Romanian healthcare. These findings are in line with previous studies developed in the European context [88,89]. Access to healthcare plays a prominent role in the population’s health status and is considered a facilitator of integration.

The design of our study allowed us to obtain insight into the participants’ views to make sense of their encounters with the healthcare system and healthcare professionals. Participants expressed both positive and negative opinions in relation to legislation and documentation.

Thus, in this study, they often cited difficulties in understanding the role of legal documentation in registering for healthcare, as they needed to have proof of identification and address upon registration with a GP. Participants showed little awareness and knowledge about legislation and did not know how to have and obtain identification documents. Failures to provide documentation can lead to delays in registering for GP services and issues in receiving secondary healthcare.

Refugees, beneficiaries of temporary protection, with short-stay rights or asylum seekers, have the right to receive free healthcare, medical treatment, and emergency medical assistance in healthcare units.

In the background of the conflict in Ukraine, when needing specialised medical assistance, those suffering from rare or chronic conditions could access emergency medical care in private unit networks such as Sanador and Medicover (unit support for Ukrainian refugee mothers with free paediatric consultations and pregnancy monitoring). Call centres were also created for free medical advice in the Ukraine language, such as Telios Care, Medicover Romania, and The National Alliance for Rare Diseases Romania in collaboration with EURORDIS—Rare Disease Europe.

On the other side of the spectrum, people that received protection according to the integration law [76] need to pay a contribution to the national health insurance, according to their income, starting with the date of obtaining that form of protection. If the persons have no taxable income, they will pay the health contribution calculated as 6.5% of the minimum national income [90]. As such, more projects need to be created and implemented to support material assistance via voucher reimbursement for rent and housing expenses, medical assistance in their enrolment to general practitioners, supplementary information for health insurance coverage, or reimbursement of medical expenses. However, Romanian authorities faced difficulties identifying the number of insured migrants and the type of insurance they benefit from [79].

Despite the fact that access to healthcare for asylum seekers is theoretically covered by health insurance, they still show little awareness and knowledge related to legislation and have difficulties understanding how the Romanian healthcare system functions.

According to Article 17(1) of Law No. 122/2006 on asylum in Romania, asylum seekers have the right to receive free-of-charge primary healthcare and appropriate treatment, emergency hospital care, as well as free-of-charge medical care and treatment in the case of acute or chronic diseases that put their lives in imminent danger, through the system of emergency medical care and qualified first aid, and the right to be included in national public health programmes aimed at the prevention, surveillance, and control of communicable diseases, in situations of epidemiological risk, i.e., the right to receive appropriate medical care during the asylum procedure [77].

Asylum seekers are assigned a personal identification number, which appears on their temporary identity documents, in order for them to benefit from all the rights provided by the law. After receiving the personal identification number, asylum seekers may register in the public health insurance system, and if they pay their healthcare contributions and register at a general practitioner’s office, they have the status of an insured person with the same rights and benefits as nationals.

This study also reveals a lack of knowledge about the cost coverage and a problematic understanding of the healthcare system’s functioning on the part of asylum seekers and people with refugee backgrounds. In most European countries (the UK, Netherlands, Spain, and Portugal), the general practitioner (GP)/family doctor functions as a ‘gatekeeper’ to the healthcare system. Barriers may arise from the gatekeepers’ lack of knowledge and cultural competence in dealing with refugee clients [60] or failure to effectively explain how the health systems are structured and what are people’s entitlements [56,57].

We have also distinguished a personal narrative type related to the ‘trust’ in healthcare professionals. Negative and positive changes in trust in healthcare professionals represent a prominent theme that could be expanded and developed in further research by using focus groups with refugees. The trust-building process is sophisticated and is not based on personal experiences but rather on stories heard and passed down from others [91].

Cultural and linguistic barriers can be overcome by creating a network of interpreters and translators, but using them is somewhat tricky since, for uncommon languages, they might not be available for emergencies. However, when interpreters and patients are from the same community, a biased situation can appear [92].

Cultural barriers might particularly have an impact on medical compliance. Previous studies demonstrated that the lack of a common language between staff and patients is associated with decreased symptom reporting and fewer referrals to secondary and specialist care [41]. The lack of language skills to navigate the healthcare systems seems to be the prevalent barrier mentioned by researchers [61,62,63,92,93,94,95,96].

## 6. Conclusions

Despite the improved efforts of Romania in tackling the sensitive issue of immigration, it has been more difficult to obtain broader support for assessing barriers faced by asylum seekers and people with refugee backgrounds. We have shared our findings with participants and public associations and sought to grasp practices, activities, and further policies.

Access to healthcare is a prominent concern that needs attention in managing the refugee situation. Even if Romania has developed procedures to facilitate the access of asylum seekers and people with refugee backgrounds to the healthcare system, most refugees experience difficulties receiving essential services in Romania; language, legislation, and finances are perceived as barriers. Cultural barriers include a lack of trust, especially towards authorities when refugees need medical assistance.

Statistics show that most asylum seekers and people with refugee backgrounds in Romania come from African and Asian countries, and only in the last half year from Ukraine. In the case of Ukrainian refugees, legislative changes and the mobilisation of civil society to provide assistance reduced possible barriers. Most of the latter category had their medical expenses covered by the state, and some were helped by NGOs.

In many situations, only NGOs offer assistance by paying for healthcare insurance and supporting some asylum seekers and people with refugee backgrounds.

Findings suggest the existence of sometimes-insurmountable gaps between current services and refugees’ needs. In the future, more clarity in procedures and logistics that correspond to the needs of these vulnerable groups and a more open and empathetic society are aspects that can contribute to improving the management of refugees and their integration into Romanian society. Research related to access to the healthcare system for refugees and asylum seekers posits challenges in methodology and comparisons when dealing with facilitators or barriers. Further research is needed to explore the extent to which the needs of refugees with other living conditions are being accommodated.

## 7. Limitations

This study has some limitations. Being a study that is exploratory in nature, it reflects the perspective of a small number of selected participants, including refugees and asylum seekers, NGOs, and other associations. An additional limitation might be the focus on barriers related more to basic needs and less to the impact of their economic integration and resettlement policies.

Further research should examine more in-depth specific barriers, such as the factors influencing access to mental healthcare services. Trained staff working in asylum centres and refugee camps could help identify persons with psychological distress.

## Figures and Tables

**Table 1:** Participants’ demographic characteristics.

Characteristics	Number	Percentage (100%)
Total (overall)	129	100%
Male	62	48.1%
Female	67	51.9%
Age group (years)		
18–29	42	32.6%
30–45	55	42.6%
46–59	19	14.7%
60+	13	10.1%
Education level		
No schooling completed	11	8.5%
Primary education	11	8.5%
Secondary education	39	30.2%
Employment status		
Unemployed but having a work permit	16	12.4%
Unemployed and without a work permit	20	15.5%
Employed	66	51.2%
Legal status		
Asylum seeker	12	9.3%
Refugee	43	33.3%
Subsidiary protection	48	37.2%
Permanent resident	21	16.3%
Naturalised citizen	7	5.4%

**Table 2:** List of NGO representatives.

Code	Involved in Working with Refugees
A1	Head of Galați Regional Integration Center under the aegis of JRS Romania
A1	Head of ‘Aluziva Association’
A1	Head of ‘Semiluna Islamic Cultural Center Foundation’
NGO’staff	
B1	Branch manager at Ecumenical Association of Churches in Romania, Timișoara
B2	Project coordinator at Ecumenical Association of Churches in Romania, Bucharest
B3	Legal advisor at International Organisation for Migration-Bucharest
B4	Project member at Ecumenical Association of Churches in Romania, Timișoara
B5	Social assistant at ‘Terres des Hommes’

**Table 3:** Perceived barriers to accessing the healthcare system.

Type of Barriers	No	Yes		
		Sometimes	Usually	Always
Financial	68.2	58.5	21.9	19.6
Legal and structural	69.8	59.0	23.1	17.9
Language	51.9	51.6	30.7	17.7
Lack of community support	76.7	76.6	16.6	6.8

**Table 4:** Relationships between pairs of words on the first theme.

Term 1	Term 2	Correlation	Significance
Language	Support	1	0
Care	Good	0.9456	0.00035
Better	Health	0.9244	0.00012
Doctor	Time	0.8882	0.00059
Necessary	Treatment	0.8291	0.00301
Accessing	Treatment	0.8403	0.00063

Source: Data from text analysis using Voyant tool.

## Data Availability

The data presented in this study are available on request from the corresponding author.
